# A Highly Focused Antigen Receptor Repertoire Characterizes γδ T Cells That are Poised to Make IL-17 Rapidly in Naive Animals

**DOI:** 10.3389/fimmu.2015.00118

**Published:** 2015-03-23

**Authors:** Yu-Ling Wei, Arnold Han, Jacob Glanville, Fengqin Fang, Luis Alejandro Zuniga, Jacob S. Lee, Daniel J. Cua, Yueh-hsiu Chien

**Affiliations:** ^1^Department of Microbiology and Immunology, Stanford University School of Medicine, Stanford, CA, USA; ^2^Department of Medicine, Division of Gastroenterology, Stanford University School of Medicine, Stanford, CA, USA; ^3^Program in Immunology, Stanford University School of Medicine, Stanford, CA, USA; ^4^Merck Research Laboratories, Palo Alto, CA, USA

**Keywords:** γδ T cells, TCR repertoire, high throughput TCR sequencing, IL-1R^+^ γδ T cells, IL-23R^+^ γδ T cells, IL-17^+^ γδ T cells

## Abstract

Interleukin (IL)-17 plays a key role in immunity. In acute infections, a rapid IL-17 response must be induced without prior antigen exposure, and γδ T cells are the major initial IL-17 producers. In fact, some γδ T cells make IL-17 within hours after an immune challenge. These cells appear to acquire the ability to respond to IL-1 and IL-23 and to make IL-17 naturally in naïve animals. They are known as the natural Tγδ17 (nTγδ17) cells. The rapidity of the nTγδ17 response, and the apparent lack of explicit T cell receptor (TCR) engagement for its induction have led to the view that this is a cytokine (IL-1, IL-23)-mediated response. However, pharmacological inhibition or genetic defects in TCR signaling drastically reduce the nTγδ17 response and/or their presence. To better understand antigen recognition in this rapid IL-17 response, we analyzed the antigen receptor repertoire of IL-1R^+^/IL-23R^+^ γδ T cells, a proxy for nTγδ17 cells in naïve animals directly *ex vivo*, using a barcode-enabled high throughput single-cell TCR sequence analysis. We found that regardless of their anatomical origin, these cells have a highly focused TCR repertoire. In particular, the TCR sequences have limited V gene combinations, little or no junctional diversity and much reduced or no N region diversity. In contrast, IL-23R^−^ cells at mucosal sites similar to most of the splenic γδ T cells and small intestine epithelial γδ lymphocytes expressed diverse TCRs. This remarkable commonality and restricted repertoire of IL-1R^+^/IL-23R^+^ γδ T cells underscores the importance of antigen recognition in their establishment/function.

## Introduction

Interleukin (IL)-17 is an important cytokine in the inflammatory response. It induces chemokines and cytokines that mediate the maturation and release of neutrophils from the bone marrow. Neutrophil recruitment focuses the immune response at the site of infection to reduce pathogen load, and induces the subsequent phases of the inflammatory response, which primes antigen-specific αβ T cell and B cell activation and initiates the resolution program. Although both αβ T cells and γδ T cells can make IL-17, αβ T cells producing IL-17 (Th17 cells) require antigen-specific priming and a specific cytokine environment to develop. In acute infections, a rapid IL-17 response must be initiated without prior antigen exposure, and γδ T cells have been identified as the major initial IL-17 producers in infections and after immunization [reviewed in Ref. (1)].

Some naïve γδ T cells in secondary lymphoid organs undergo antigen-driven activation and differentiation to become IL-17 producers: within 24 h after immunization, antigen-specific γδ T cells in the draining lymph node increase in numbers and show activated phenotypes (e.g., becoming CD44^hi^ and CD62L^lo^). Forty-eight hours after immunization, activated γδ T cells express RORγt and after another 12 h, these cells make IL-17A and IL-17F (2, 3), these are the inducible Tγδ17 cells. Importantly, encountering antigen in an immune response induces the expression of inflammatory cytokine receptors such as IL-1R and IL-23R on γδ T cells. Signaling through the T cell receptor (TCR) and the cytokine receptors can then induce sustained, high magnitude IL-17 production (2, 4). These observations provide a mechanistic basis for the induction of a sustained antigen-specific γδ T cell IL-17 response, which is much more rapid than that of Th17 αβ T cells.

In addition to the inducible Tγδ17 cells discussed above, some γδ T cells in naïve mice, such as those in the skin dermis, the peritoneum, intestinal lamina propria, the lung, and the spleen have an activated phenotype (CD44^hi^CD62L^lo^) and express IL-1R and IL-23R. These cells make IL-17 a few hours after immune challenge-these are the natural Tγδ17 (nTγδ17) cells (1). The observation that IL-17 can be induced with IL-1 and IL-23 alone without deliberate TCR triggering has led to the supposition that the antigen recognition by these cells is irrelevant to their response (5). Nonetheless, this response is inhibited by cyclosporine A (CsA) or by FK506 (2). Both compounds reduce nuclear factor of activated T cells (NFAT) activity and disrupt the calcineurin-NFAT signaling circuit activated by signaling through the antigen receptor (6). Furthermore, the amount of IL-17 induced by the inflammatory cytokines alone is much lower in magnitude when compared with that induced by cytokines together with TCR stimulation (2, 4), suggesting that robust IL-17 production requires combined signaling through the TCR and cytokine receptors. Moreover, the number of rapid IL-17 responding IL-1R^+^ γδ T cells in the intestinal lamina propria and peritoneum is markedly reduced in germ free mice, and in SPF mice treated with the antibiotic neomycin sulfate, vancomycin but not in mice treated with metronidazole when compared with SPF mice and the numbers can be restored by SPF microbiota reconstitution. However, the presence of these IL-1R^+^ γδ T cells requires signaling through VAV1, a guanine nucleotide exchange factor required for the activation of γδ T cells via γδ TCR ligation (7), but not the myeloid differentiation primary response protein 88 (MyD88) or toll-like receptor 3 signaling pathways (8). In addition, the number of nTγδ17 cells is drastically reduced in the SKG mouse (9), which carries a mutation that reduces the function of the kinase domain of the TCR-proximal signaling kinase Zap70. These observations demonstrate the importance of TCR signaling in nTγδ17 induction and function. To evaluate the contribution of antigen recognition to their function, we seek to determine the antigen receptor repertoire of nTγδ17 cells. To this end, we use a bar-code-enabled high throughput single-cell TCR sequencing strategy, which allows us to identify the TCR γ and δ gene pair from each cell directly *ex vivo*, without the bias introduced through generating T cell clones or hybridomas. This method determines the entire sequence of both the TCR γ and δ chains, including the V gene segment and CDR3 region, such that we can properly define the antigen receptor specific repertoire, rather than describing these cells solely based on their Vγ or Vδ usage. The results are discussed below.

## Materials and Methods

### Mice

C57BL/6 mice were purchased from Jackson Laboratories and housed in the Stanford Animal Facility for at least 1 week before use. IL-17F^Thy1.1/Thy1.1^ mice (10) were bred and housed in the pathogen-free Stanford Animal Facility. IL-23R EGFP mice (11) were bred and housed in the pathogen-free Merck Research Laboratories, Palo Alto Animal Facility. All experiments were performed in accordance with the Institutional Biosafety Committee and the Institutional Animal Care and Use Committee.

### Antibodies and cell isolation

Antibodies were purchased from either eBioscience or BD Biosciences unless otherwise stated. All analyses and sorting were performed on a BD Aria or Falstaff sorter. γδ T cells were enriched from mouse splenocytes or peritoneal cells by negative depletion as described (2).

To isolate Thy1.1 positive spleen γδ T cells from IL-17F*^Thy1.1/Thy1.1^* reporter mice, enriched γδ T cells were stained with PE-GL3, Pacific Blue-CD3e, PerCPCy5.5-Thy1.1, PerCP/Cy5.5 Mouse IgG1, κ Isotype Ctrl (OX-7 and its isotype control; BioLegend), LIVE/DEAD Aqua, APC-Cy7 conjugated anti-TCRβ, CD19, CD11b, CD11c, F4/80, TER-119. APC-Cy7 and Aqua positive cells are excluded from analysis. Peritoneal IL-1R positive γδ T cells were isolated from C57BL/6J mice i.p. infected with 1000 tachyzoites of Type II Me49 strain of *Toxoplasma gondii* 5 h prior. To isolate IL-1R (CD121a) positive cells, enriched γδ T cells were stained with PE-GL3 (pan anti-γδ TCR), PE-Cy7-CD3e (145-2C11), APC-CD121a (JAMA-147; BioLegend), LIVE/DEAD Aqua, and APC-Cy7 conjugated anti-TCRβH57-597), CD19 (1D3), CD11b (M1/70), CD11c (N418), F4/80 (BM8), TER-119 (TER-119). APC-Cy7 and Aqua positive cells are excluded from analysis. Dermal split-thickness skin was obtained from C57BL/6J mice ears. Dermal sheets were prepared by incubation of split-thickness skin with 0.25% trypsin for 16 h at 4°C, and subsequent removal of the epidermis. Dermal sheets were digested with 2.5 mg/ml collagenase and 0.3 mg/ml hyaluronidase for 45 min at 37°C to release dermal cells. Dermal cells were stained with PE-GL3, APC-Cy7-CD3e antibodies and Live/Dead Aqua. GL3 and CD3e positive dermis γδ T cells were isolated with FACS.

Two- to four-month-old female IL-23R EGFP^+/−^ mice were used for the isolation of IL-23R^+^ and IL23R^−^ γδ T cells. Five mice were combined for each type of tissue preparation. Visceral fat was directly minced in 4 mg/ml collagenase II (Worthington), 5% FBS in RPMI followed by shaking for 45 min at 37°C. Cells were further purified with 36% Percoll gradient (GE Healthcare) in PBS and spun at 2000 rpm for 5 min at room temperature. The floating layer and Percoll layer were aspirated and the resulting cell pellet was suspended in PBS, counted, and stained for flow cytometry. Colons were cleaned and washed in PBS and minced into 1 cm segments and placed into 0.5 mM EDTA in PBS. After shaking for 20 min at 37°C, the intraepithelial cell rich supernatant was discarded. Colon fragments were washed with PBS, then further minced to pieces <0.25 cm^3^ in size in digestion buffer [PBS + 10% FCS + 1 mg/ml collagenase D (Sigma) + 2000 U/ml DNase I (Sigma) + Dispase (Corning, dilute 1:100)], and incubated with shaking for 20 min at 37°C. Cells were further purified with percoll gradient as described for isolating cells from fat. Isolated cells were stained with FcBlock, CD3 Percp-Cy5.5, TCRδ APC (Clone GL3), TCRβ APC-Cy7, CD4-PE, CD8α PE-Cy7, Live/Dead Aqua. IL-23R GFP^+^ and IL-23R GFP^−^ γδ T cells were single sorted into the wells of a 96-well plate using a FACsAria II (BD Biosciences).

### Barcode-enabled high throughput single-cell TCR determination

Single T cells are sorted into 96-well PCR plates and sequencing is performed as described (12), except murine γδ TCR specific primers are used for this study. γδ TCR primer sequences and the sequencing reaction are described in detail in Supplemental Methods in Supplementary Material. Briefly, an RT-PCR reaction is carried out with TCR primers. The products are then used in a second PCR reaction, with nested primers for TCR genes. A third reaction is then performed that incorporates individual barcodes into each well. The products are combined, purified, and sequenced using the Illumina MiSeq platform. The resulting paired-end sequencing reads are assembled and de-convoluted using barcode identifiers at both ends of each sequence by a custom software pipeline to separate reads from every well in every plate. The resulting sequences are analyzed using VDJFasta (13), which we have adapted to resolve barcodes and analyze sequences with a customized gene-segment database. The CDR3 nucleotide sequences are then extracted and translated. Barcode design is shown in Figure S1 in Supplementary Material and TCR sequencing primer sequences are shown in Table S2 in Supplementary Material.

## Results

**Figure 1:**
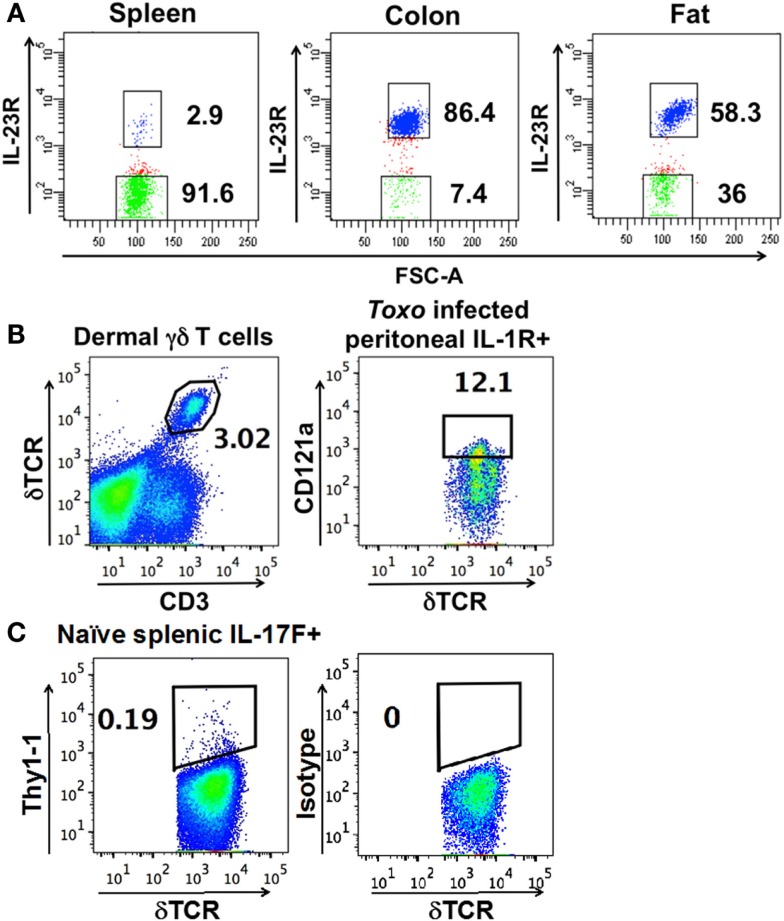
**Representative FACS analysis and gates used to isolate (A) IL-23R^+^ (in blue) and IL-23R^−^ γδ T cells (in green) (using FACSDiva) from IL-23R reporter mice**. **(B)** Dermal cells, IL-1R^+^ γδ T cells from the peritoneum of C57BL/6 mice infected with *T. gondii* 5 h prior. **(C)** Thy1.1^+^ cells from the spleen of naïve IL-17F reporter mice. **(B,C)** are plotted using FlowJo. The number within each graph indicates the percentage of the designated population of cells out of the total γδ T cells.

**Figure 2:**
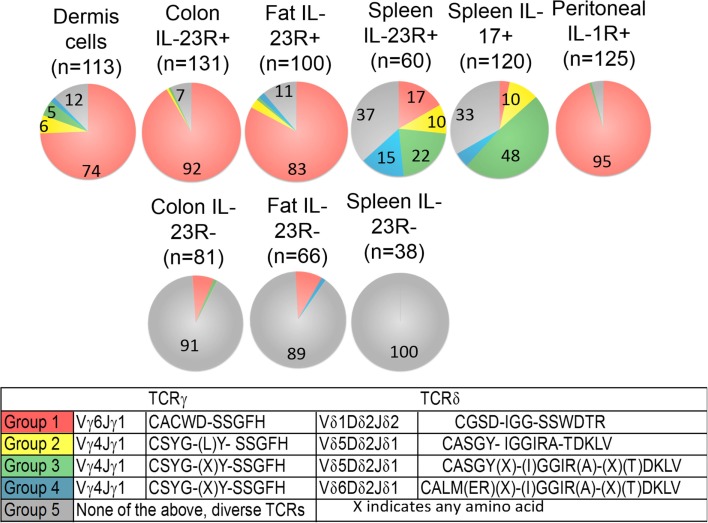
**Frequency of each major group of TCR sequences in IL-1R^+^/IL23R^+^ γδ T cell populations**. Spleen IL-17^+^, IL-23R^+^, IL-23R^−^ γδ T cells, lung, fat, and colon lamina propria IL-23R^+^ and IL-23R^−^ γδ T cells, peritoneum IL-1R^+^ γδ T cells 5 h after intraperitoneum *Toxoplasma gondii* infection and skin dermal γδ T cells were analyzed. Each cell population is represented by one pie chart. Each section of the pie chart represents one group of TCR sequences, color-coded as described. *n*, total number of analyzed sequences. The number within each section of the pie chart indicates the percentage of a given group of TCR sequences in the total number of analyzed sequences of that cell population (Table S1 in Supplementary Material). All experiments were performed two independent times, except the analysis of spleen IL-23R^+^ and IL-23R^−^ γδ T cells, which were isolated and analyzed once. TCR sequences from two independent isolations and analyses are very similar and the combined results are shown. In two independent experiments, 58% and 82% of the total colon γδ T cells are IL23R^+^; 74% and 86% of total fat γδ T cells are IL-23R^+^; 0.1% and 0.2% of spleen cells are IL-17F^+^; 2.9% of spleen γδ T cells are IL23R^+^. In the peritoneum 5 h after infection, 12 and 30% of the γδ T cells are IL-1R^+^.

A defining feature of nTγδ17 cells is their surface expression of IL-1R and IL-23R in naïve animals. To determine the antigen receptor repertoire of γδ T cells that are “poised” to mount a rapid IL-17 response, we analyzed skin dermal cells, and IL-23R^+^ γδ T cells in the colon lamina propria, fat, and spleen of naïve IL-23R reporter mice (IL-23R EGFP). Peritoneal nTγδ17 cells are characterized by their IL-1R expression in rapid response situations (8); therefore, we analyzed IL-1R^+^ peritoneal γδ T cells from C57/BL6 mice that were intra-peritoneally (i.p.) infected with *T. gondii* 5 h prior. Representative FACS analysis and gates used to isolate these cells are shown in Figure 1. The TCR sequences were determined from a single FACS sorted γδ T cell using a bar-code-enabled high throughput single-cell TCR sequencing strategy. We found that IL-17F^+^ spleen γδ T cells from naïve IL-17F reporter mice (Il17f thy1.1/thy1.1) and IL-23R^+^ spleen γδ T cells from naïve IL-23R reporter mice have similar TCR repertoires (Figure 2). This observation is consistent with the supposition that IL-23R^+^ γδ T cells in naïve animals can be used as a proxy for nTγδ17 cells in TCR repertoire analysis.

A striking characteristic of the TCR repertoire of IL-1R^+^/IL-23R^+^ γδ T cells is the lack of diversity. They express TCRs with limited V gene combinations, little or no junctional diversity and much reduced or no N region diversity. In particular, a single pair of TCR sequences encoded by Vδ1Dδ2Jδ1 and Vγ6Jγ1 (Group 1 sequences, Figure 2) dominates the repertoire of dermal cells, IL-23R^+^ γδ T cells from the lung, colon, and IL-1R^+^ γδ T cells from the peritoneum. These cells also utilize two sets of closely related TCR sequences, which consist of similar Vγ4Jγ1 rearrangements, paired with very similar Vδ5Dδ2Jδ2 (designated as Group 2, 3 sequences, Figure 2). Naïve spleen IL-23R^+^ and IL-17F^+^ T cells did not have a dominant population that expressed Group 1 sequences. Instead, cells with the Group 3 sequences were more represented. Some of these γδ T cells also expressed TCRs consisting of Group 3 TCRγ chains paired with a very similar Vδ4Dδ2Jδ2 TCRδ chains (designated as the Group 4 sequences, Figure 2).

**Figure 3:**
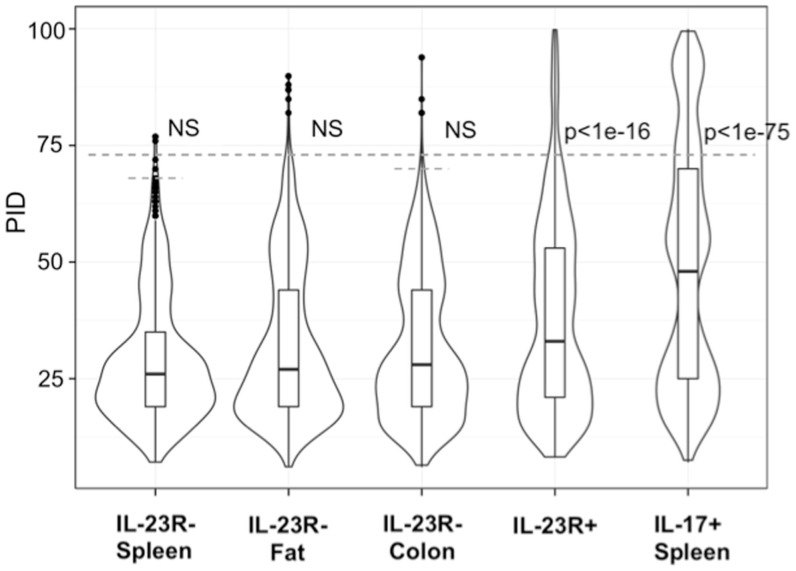
**Analysis of CDR3 paratope convergence of all unique sequences of IL-23R^−^ γδ T cells from the spleen, fat, colon samples, IL-17F^+^ γδ T cells from spleen and IL-23R^+^ γδ T cells (combined from all anatomical sites)**. Percent identity among aligned γ and δ CDR3 amino acid sequences for all pairwise comparisons within each group are represented in violin/Box plot. Significance assessed by the Mann–Whitney–Wilcoxon test with Bonferroni’s correction for multiple testing given *a* = 0.01 set to *p* < 0.001 to be considered significance. Dotted line indicates average 99th percentile percent identity for the IL-23R^−^ T cell populations (68% ID for spleen, 73% ID for fat, 70% ID for colon).

In contrast, reported TCR sequences identified from spleen γδ T cells and small intestine epithelial γδ lymphocytes (IELs) (14–16) and IL-23R^−^ γδ T cell populations in the spleen, lung, and colon lamina propria analyzed here (Table S1 in Supplementary Material) are highly diverse, using different Vγ’s and Vδ’s, with CDR3 regions consisting of both Dδ1 and Dδ2 gene segments in all three reading frames, and N regions in each of the gene-segment junctions. An analysis of CDR3 paratope convergence within IL-23R^−^, IL-23R^+^, and lL-17F^+^ γδ T cell populations is shown in Figure 3. Along this line, it should be noted that the antigen-specific γδ T cells, including the inducible Tγδ17 cells, also utilize diverse TCRs (2, 3, 16). In this context, ~1/3 of the IL-23R^+^ or IL-17F^+^ spleen γδ T cells, and ~1/5 of IL-23R^+^ lung γδ T cells express TCRs with different VγVδ genes and diverse CDR3 regions. The spleen and lungs are continuously exposed to blood-borne or air-borne environmental antigens. It is likely that the TCR repertoire of IL-1R^+^/IL-23R^+^ γδ T cells reflects both the natural and the inducible Tγδ17 cells.

**Figure 4:**
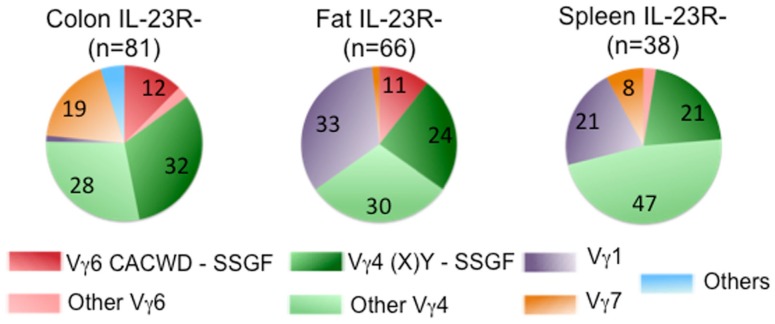
**Frequency of Vγ chain usage in IL-23R^−^ cell population**. Each pie chart represents each cell population. *n*, total number of analyzed sequences. Number with each section of the pie chart, the percentage of each group of sequences color-coded as indicated in Figure 2.

Despite the fact that a substantial number of IL-1R^+^/IL-23R^+^ γδ T cells and dermal γδ T cells express TCRs with similar Vγ4Jγ1 rearrangement (CSYG-(X)Y-SSGFHK), Vγ4^+^ TCRγ chain sequences are not utilized exclusively by this set of T cells. In fact, ~50% of the IL-23R^**−**^ cells also expressed TCRs with Vγ4, and more than half of these Vγ4 sequences were also expressed in IL-23R^+^ cell populations (Figure 4).

## Discussion

Our analysis showed that regardless of their anatomical location, IL-1R^+^/IL-23R^+^ γδ T cells express a highly focused antigen receptor repertoire. While all major groups of TCR sequences expressed by these cells result from rearrangements with exonuclease digestion and P nucleotide addition (17), only Group 3 and 4 TCR sequences have N nucleotides at the CDR3 γ and δ junctions. The N nucleotides are generated at the terminal of the combining gene segments by terminal transferase (TdT) in a template-independent manner. In mice, TdT is not expressed in developing thymocytes until 4–5 days after birth (18). Thus, γδ T cells that express Group 1 and 2 sequences are most likely generated during the fetal and/or neonatal stages. Indeed, Group 1 TCR has also been described for hybridomas derived from fetal and newborn γδ thymocytes (19) and is also present at the mucosal sites (20–22). Our observation that Group 3, 4 TCR expressing IL-1R^+^/IL-23R^+^ γδ T cells are prevalent in the spleen and present in the lung and skin is consistent with the observation that adult precursor cells contribute to the nTγδ17 cell pool and that these cells express Vγ4^+^ TCR γ chains (23–25).

Group 1 TCR sequences have been described for γδ T cell hybridomas generated from lung epithelium (26), from expanded γδ T cells after *Listeria monocytogenes* and *Bacillus subtilis* infection and in models of autoimmune inflammation (27–29). In addition, the rapid appearance of Vγ6 and/or Vδ1 Tγδ17 cells has been reported in various infection systems: *E. coli* (i.p.) (30, 31), *L. monocytogenes* (i.p. oral) (32, 33) and *Staphylococcus aureus* (i.p.) (34). Vγ6^+^ and Vγ4^+^ dermal γδ T cells making IL-17 in response to imiquimod applied topically to induce skin inflammation has also been reported (24, 25). Separated TCR γ and δ chains of Group 4 sequences were identified from CFA-induced IL-17 making γδ T cells (35, 36). Taken together, our repertoire analysis confirms and advances previous studies of TCR usage of nTγδ17 cells by defining the precise TCR sequences of these cells and observing how constrained they are. These observations suggest that antigen encountering is important for establishing their functional attributes, a finding consistent with observations that signaling through the TCR is essential for this process (2, 8, 9).

It is unclear what nTγδ17 cells recognize. However, the identification of their TCR sequences is an important step forward in characterizing the antigens of these cells. In this context, O’Brien, Born and their colleagues demonstrated that a multimeric staining reagent of soluble TCR expressing the Group 1 sequences can bind L cells, NIH 3T3 cells, a keratinocyte cell line XB-2, as well as freshly isolated macrophages from naïve mice and from mice infected with *Listeria* (37, 38).

While nTγδ17 responses are well documented in the mouse, it is unclear whether or not a human counterpart exists. In this regard, human and murine γδ TCR gene sequences are very different. Thus, it is unlikely that one would find human γδ TCRs that show the sequence equivalent of the TCRs described for the murine nTγδ17 cells. However, one of the defining characteristics of adaptive immune recognition is that the antigen specificity, but not the particular antigen-specific receptor sequences, is conserved through evolution. The recognition of lysozyme by specific murine, human, and camel antibodies as well as by the adaptive immune receptors of sea lamprey (39), and the recognition of the algae protein phycoerythrin (PE) by specific human and murine γδ TCRs (2) are such examples. Thus, differences in the TCR gene sequences among different species should not preclude the presence of nTγδ17 cells.

It should be noted that the focused antigen receptor repertoire described here is based on the analysis of pairs of TCR γ and δ chains, consisting of V gene segments as well as CDR3 regions. While the majority of these γδ T cells expressed Vγ6 or Vγ4, not all Vγ6 and Vγ4 expressing cells belong to this group of nTγδ17 cells. These observations underscore the need for caution in categorizing γδ T cell function solely according to V gene usage. The approach of determining TCR sequences from a single cell directly *ex vivo*, as outlined here, should facilitate future analysis of the contributions of γδ T cells to a range of immune responses.

## Conflict of Interest Statement

The authors declare that the research was conducted in the absence of any commercial or financial relationships that could be construed as a potential conflict of interest.

## Supplementary Material

The Supplementary Material for this article can be found online at http://journal.frontiersin.org/article/10.3389/fimmu.2015.00118
