# Supplementary material for: A Highly Focused Antigen Receptor Repertoire Characterizes γδ T Cells That are Poised to Make IL-17 Rapidly in Naive Animals
Source: Front Immunol. 2015 Mar 23;6:118. doi: 10.3389/fimmu.2015.00118 (PMC4370043; doi:10.3389/fimmu.2015.00118)
Supplement: Supplementary file 1 [file Data_Sheet_1.ZIP › Figure S1.PDF]

Supplementary Figure 1

a

Enables amplification by Illumina^{TM} paired-end Primers

Amplification of common Sequence from PCR reaction #2

CCTACACGACGCTCTTCCGATCTNN**XXXXX**GA**XXXXX**CCAGGGTTTTCCCAGTCACGAC

Plate barcode

1. GCAGA
2. TCGAA
3. AACAA
4. GGTGC
5. TTGGT
6. CATTC
7. ATTGG
8. CGGTT
9. ATCCT
10. ATGTC
11. TCACG
12. AGACC
13. CCCCA
14. GCGCT
15. TCCTT
16. TATAT
17. CGTAA
18. AAGGT
19. AGCTC
20. CTTGC
21. GTATC
22. TATGA
23. CACAC
24. ACACT
25. ACTAC

Row barcode

A. TAAGC
B. TGCAC
C. CTCAG
D. GGAAT
E. CGAGG
F. AGGAG
G. TGTTG
H. CAACT

b

Enables amplification by Illumina^{TM} paired-end Primers

Amplification of constant regions (TCR genes) or common sequence for phenotyping genes

CTGCTGAACCGCTCTTCCGATCTNN**XXXXX**CACCAGACAAGCAACATTTGTTCC (TCR delta)
GATTTGTTTCAGCAACAGAAGGAAG (TCR gamma 1-3)
GGAGATTTGTTTCAGCAGCAGAAG (TCR gamma 4)

Column barcode

1. GTTCA
2. CAGGA
3. TTATA
4. CCTGT
5. ACCGC
6. ACTTA
7. GCTAG
8. GACGT
9. GGCTA
10. GAATG
11. CCAAC
12. GAGAC

Barcoding primer design. (**a**) 5' primers incorporate barcodes that specify plate and row and bind and amplify a common sequence that is incorporated into all 5' primers from PCR reaction #2. The outside sequence allows for amplification using IlluminaTM Paired-End primers. (**b**) 3' primers incorporate barcodes that specify column. The primers amplify nested constant region sequences for TCRγ/δ. The outside sequence allows for amplification using IlluminaTM paired-end primers.
