# Supplementary material for: A Highly Focused Antigen Receptor Repertoire Characterizes γδ T Cells That are Poised to Make IL-17 Rapidly in Naive Animals
Source: Front Immunol. 2015 Mar 23;6:118. doi: 10.3389/fimmu.2015.00118 (PMC4370043; doi:10.3389/fimmu.2015.00118)
Supplement: Supplementary file 1 [file Data_Sheet_1.ZIP › Supplemental Methods.PDF]

Supplemental Methods

γδ TCR Sequencing

Single-cell sorting was performed using an ARIA II cell sorter (Becton Dickinson). γδ TCR sequences from single cell were obtained by a series of three nested PCR reactions as described (1). Briefly, cells were sorted directly into RT-PCR buffer. For the first reaction, reverse transcription and pre-amplification are performed with a One-Step RT-PCR kit (Qiagen) using multiplex PCR with multiple Vγ and Vδ region primers, one Cδ and two Cγ region primers in a 12 μl reaction. For the RT-PCR reaction #1, the final concentration of each TCR V region primer is 0.36 μM, each C region primer is 0.6 μM. A 25 cycle first RT-PCR reaction was performed per manufacturer's instructions using the following cycling conditions: 50° 30'; 95° 15'; 94° 30", 62° 1', 72° 1' x 25 cycles; 72° 5'; 4°. Next, a 1 μl aliquot of the first reaction was used as a template for second 12 μl PCR using HotStarTaq DNA polymerase (Qiagen). The following cycling conditions were: 95° 15'; 94° 30", 64° 1', 72° 1' x 25 cycles. For the TCR sequencing reaction, multiple internally nested TCRVγ, TCRVδ, TCRCγ and Cδ primers (Supplementary Table **1**) were used (V primers 0.36 μM, C primers 0.6 μM). The second set of TCRV region primers contained a common 23 base sequence at the 5' end to enable further amplification (during the third reaction) with a common 23 base primer. 1 μl aliquot of the second PCR product was used as a template for the third 14 μl PCR reaction, which incorporates barcodes and enables sequencing on the Illumina™ MiSeq™ platform[1]. For the third and final PCR reaction for TCR sequencing, amplification was performed with HotStarTaq DNA polymerase for 36 cycles using a 5' barcoding primer (0.05 μM) containing the common 23 base sequence and a 3' barcoding primer (0.05 μM) containing sequence of a third internally nested Cγ and/or Cδ primer, and Illumina™ Paired-End primers (0.5μM each). For TCRγ amplification, two sets of 3' barcoding primers are used to enable amplification of all TCRγ C-regions. In addition to the common 23 base sequence at the 3' end (that enables amplification of products from the second reaction) and a common 23 base sequence at the 5' end (that enables amplification with Illumina Paired-End primers), each 5' barcoding primer contains a unique 5 base barcode that specifies plate and a unique 5 base barcode that specifies row within the plate[1]. These 5' barcoding primers were added with a multichannel pipette to each of 12 wells within a particular row within a particular plate. In addition to the internally nested TCR C-region sequence and a common 23 base sequence at the 3' end (that enables amplification with Illumina™ Paired-End primers), each 3' barcoding primer contains a unique 5-nucleotide barcode that specifies column. These 3' barcoding primers were added with a multichannel pipette to each of 8 wells within a column within all plates. After the third and final PCR reaction, each PCR product should have a unique set of barcodes incorporated that specifies plate, row and column and have Illumina™ Paired-End sequences that enable sequencing on the Illumina™ MiSeq™ platform. The

PCR products were combined at equal proportion by volume, run on a 1.2% agarose gel, and a band around 350 to 380 bp was excised and gel purified using a Qiaquick gel extraction kit (Qiagen). This purified product was then sequenced.

1 Han, A., Glanville, J., Hansmann, L. & Davis, M. M. Linking T-cell receptor sequence to functional phenotype at the single-cell level. *Nature biotechnology* **32**, 684-692, doi:10.1038/nbt.2938 (2014).
