# Supplementary material for: A Highly Focused Antigen Receptor Repertoire Characterizes γδ T Cells That are Poised to Make IL-17 Rapidly in Naive Animals
Source: Front Immunol. 2015 Mar 23;6:118. doi: 10.3389/fimmu.2015.00118 (PMC4370043; doi:10.3389/fimmu.2015.00118)
Supplement: Supplementary file 1 [file Data_Sheet_1.ZIP › Table S2.PDF]

Reaction 1

mouseDV1	GGAGGAGATGAGAAAAGTAAGGAC
mouseDV2	CCAAGAAGCATACAAGCAGTATAATG
mouseDV4	GCAATTCTACTGATGGTGGAAAGAG
mouseDV5	CCCATGATGCAGATTTTGTTCAAGG
mouseDV6	TAGTGGAGAGATGGTTTTSCTTATT
mouseDV7	GGAAGMCTCGTCAGCCTGTTGT
mouseDV8	CAGTGTCCGATAAAAAGGAAGATGGA
mouseDV9	AGAGCCTCAAGGGACAAAGAGAAA
mouseDV10	CTTGGCTTCAGGAACAAAGGAGAA
mouseDV11	CTCTTACAAAAAGGAAAATGCAACAG
mouseDV12	GGTCTACAACAAAATACAACCAATAG
mouseGV1-3	CAATCAACRACCCTTAGRAGGGAAG
mouseGV4	ATGTCCTTGCAACCCCTACCCAT
mouseGV5	CAGTCAAAACTTACAAACAAGACAAGT
mouseGV6	CCAAAGAATGCTGTGTAGTTCTTCAA
mouseGV7	CCACAACTGTGGTGGATTCCAGAT
mouseDC	CCACAATCTTCTTGGATGATCTGAG
mouseGC1-3	GGAAAGAACTTTTCAAGGASACAAAG
mouseGC4	CCCTTATGACTTCAGGAAAGAACTTT

Reaction 2

mouseDV1	CCAGGGTTTTCCCAGTCACGACnnCGCTAAGCTGGATAAGAAAATGCAG
mouseDV2	CCAGGGTTTTCCCAGTCACGACnnCTCTGTGAACTTCCAGAAAGCAGC
mouseDV4	CCAGGGTTTTCCCAGTCACGACnnCCTCAAAGGGAAAATTAACATTTCAAA
mouseDV5	CCAGGGTTTTCCCAGTCACGACnnCGATTTTCTGTGAAGCACAGCAAG
mouseDV6	CCAGGGTTTTCCCAGTCACGACnnCTAYTCTGTAGTCTTCCAGAAATCA
mouseDV7	CCAGGGTTTTCCCAGTCACGACnnGTMCAATCCTTCTGGGACAAAGCA
mouseDV8	CCAGGGTTTTCCCAGTCACGACnnGATTCACAATCTTCTTCAATAAAAGGGA
mouseDV9	CCAGGGTTTTCCCAGTCACGACnnGGAAGCAGCAGAGGKTTTGAAGC
mouseDV10	CCAGGGTTTTCCCAGTCACGACnnGGGAGGCTAAAGTCAGCATTTGAT
mouseDV11	CCAGGGTTTTCCCAGTCACGACnnGGTCATTATTCTCTGAACTTTCAGAAG
mouseDV12	CCAGGGTTTTCCCAGTCACGACnnGGCTATTGCCTCTGACAGAAAGT
mouseGV1-3	CCAGGGTTTTCCCAGTCACGACnnACAARAAAATTGAAGCAAGTAAAGATTTT
mouseGV4	CCAGGGTTTTCCCAGTCACGACnnCTTAGATAAGGAGTACAAGAAAATGGA
mouseGV5	CCAGGGTTTTCCCAGTCACGACnnCCACTCCCGCTTGGAAATTGATGA
mouseGV6	CCAGGGTTTTCCCAGTCACGACnnGTGACGAAAGATATGAGGCAAGGA
mouseGV7	CCAGGGTTTTCCCAGTCACGACnnCATGTTTATGAAGGCCCGGACAAGA
mouseDC	GTCACCTCTTTAGGGTAGAAATCTT
mouseGC1-3	ACAAAGGTATGTCCCAGTCTTATGGA
mouseGC4	GGAGACAAAGGTAGGTCCCAGC

Reaction 3

mouseDCbc1	CTGCTGAACCGCTCTTCCGATCTnnGTTCACACCAGACAAGCAACATTTGTTCC
mouseDCbc2	CTGCTGAACCGCTCTTCCGATCTnnCAGGACACCAGACAAGCAACATTTGTTCC
mouseDCbc3	CTGCTGAACCGCTCTTCCGATCTnnTTATACACCAGACAAGCAACATTTGTTCC
mouseDCbc4	CTGCTGAACCGCTCTTCCGATCTnnCCTGTCACCAGACAAGCAACATTTGTTCC
mouseDCbc5	CTGCTGAACCGCTCTTCCGATCTnnACCGCCACCAGACAAGCAACATTTGTTCC
mouseDCbc6	CTGCTGAACCGCTCTTCCGATCTnnACTTACACCAGACAAGCAACATTTGTTCC
mouseDCbc7	CTGCTGAACCGCTCTTCCGATCTnnGCTAGCACCAGACAAGCAACATTTGTTCC
mouseDCbc8	CTGCTGAACCGCTCTTCCGATCTnnGACGTCACCAGACAAGCAACATTTGTTCC
mouseDCbc9	CTGCTGAACCGCTCTTCCGATCTnnGGCTACACCAGACAAGCAACATTTGTTCC
mouseDCbc10	CTGCTGAACCGCTCTTCCGATCTnnGAATGCACCAGACAAGCAACATTTGTTCC
mouseDCbc11	CTGCTGAACCGCTCTTCCGATCTnnCCAACCACCAGACAAGCAACATTTGTTCC
mouseDCbc12	CTGCTGAACCGCTCTTCCGATCTnnGAGACCACCAGACAAGCAACATTTGTTCC
mouseGC1-3bc1	CTGCTGAACCGCTCTTCCGATCTnnGTTCAGATTTGTTTCAGCAACAGAAGGAAG
mouseGC1-3bc2	CTGCTGAACCGCTCTTCCGATCTnnCAGGAGATTTGTTTCAGCAACAGAAGGAAG
mouseGC1-3bc3	CTGCTGAACCGCTCTTCCGATCTnnTTATAGATTTGTTTCAGCAACAGAAGGAAG
mouseGC1-3bc4	CTGCTGAACCGCTCTTCCGATCTnnCCTGTGATTTGTTTCAGCAACAGAAGGAAG
mouseGC1-3bc5	CTGCTGAACCGCTCTTCCGATCTnnACCGCGATTTGTTTCAGCAACAGAAGGAAG
mouseGC1-3bc6	CTGCTGAACCGCTCTTCCGATCTnnACTTAGATTTGTTTCAGCAACAGAAGGAAG

mouseGC1-3bc7	CTGCTGAACCGCTCTTCCGATCTnnGCTAGGATTTGTTTCAGCAACAGAAGGAAG
mouseGC1-3bc8	CTGCTGAACCGCTCTTCCGATCTnnGACGTGATTTGTTTCAGCAACAGAAGGAAG
mouseGC1-3bc9	CTGCTGAACCGCTCTTCCGATCTnnGGCTAGATTTGTTTCAGCAACAGAAGGAAG
mouseGC1-3bc10	CTGCTGAACCGCTCTTCCGATCTnnGAATGGATTTGTTTCAGCAACAGAAGGAAG
mouseGC1-3bc11	CTGCTGAACCGCTCTTCCGATCTnnCCAACGATTTGTTTCAGCAACAGAAGGAAG
mouseGC1-3bc12	CTGCTGAACCGCTCTTCCGATCTnnGAGACGATTTGTTTCAGCAACAGAAGGAAG
mouseGC4bc1	CTGCTGAACCGCTCTTCCGATCTnnGTTCAGGAGATTTGTTTCAGCAGCAGAAG
mouseGC4bc2	CTGCTGAACCGCTCTTCCGATCTnnCAGGAGGAGATTTGTTTCAGCAGCAGAAG
mouseGC4bc3	CTGCTGAACCGCTCTTCCGATCTnnTTATAGGAGATTTGTTTCAGCAGCAGAAG
mouseGC4bc4	CTGCTGAACCGCTCTTCCGATCTnnCCTGTGGAGATTTGTTTCAGCAGCAGAAG
mouseGC4bc5	CTGCTGAACCGCTCTTCCGATCTnnACCGCGGAGATTTGTTTCAGCAGCAGAAG
mouseGC4bc6	CTGCTGAACCGCTCTTCCGATCTnnACTTAGGAGATTTGTTTCAGCAGCAGAAG
mouseGC4bc7	CTGCTGAACCGCTCTTCCGATCTnnGCTAGGGAGATTTGTTTCAGCAGCAGAAG
mouseGC4bc8	CTGCTGAACCGCTCTTCCGATCTnnGACGTGGAGATTTGTTTCAGCAGCAGAAG
mouseGC4bc9	CTGCTGAACCGCTCTTCCGATCTnnGGCTAGGAGATTTGTTTCAGCAGCAGAAG
mouseGC4bc10	CTGCTGAACCGCTCTTCCGATCTnnGAATGGGAGATTTGTTTCAGCAGCAGAAG
mouseGC4bc11	CTGCTGAACCGCTCTTCCGATCTnnCCAACGGAGATTTGTTTCAGCAGCAGAAG
mouseGC4bc12	CTGCTGAACCGCTCTTCCGATCTnnGAGACGGAGATTTGTTTCAGCAGCAGAAG

PEprimer1	AATGATACGGCGACCACCGAGATCTACACTCTTTCCCTACACGACGCTCTTCCGATCT
PEprimer2	CAAGCAGAAGACGGCATACGAGATCGGTCTCGGCATTCCTGCTGAACCGCTCTTCCGATCT
